# Transfer free graphene growth on SiO_2_ substrate at 250 °C

**DOI:** 10.1038/srep43756

**Published:** 2017-03-02

**Authors:** Riteshkumar Vishwakarma, Mohamad Saufi Rosmi, Kazunari Takahashi, Yuji Wakamatsu, Yazid Yaakob, Mona Ibrahim Araby, Golap Kalita, Masashi Kitazawa, Masaki Tanemura

**Affiliations:** 1 Nagoya Institute of Technology, Department of Frontier Materials, Graduate School of Engineering, Gokiso-cho, Showa-ku, Nagoya 466-8555, Japan; 2 Universiti Pendidikan Sultan Idris, Department of Chemistry, Faculty of Science and Mathematics, 35900 Tanjong Malim, Perak, Malaysia; 3 Universiti Putra Malaysia, Department of Physics, Faculty of Science, 43400 UPM Serdang, Selangor, Malaysia; 4 Olympus Co. Ltd., 6666 Inatomi, Tatsuno, Kami-Ina-Gun, Nagano 399-0495, Japan

## Abstract

Low-temperature growth, as well as the transfer free growth on substrates, is the major concern of graphene research for its practical applications. Here we propose a simple method to achieve the transfer free graphene growth on SiO_2_ covered Si (SiO_2_/Si) substrate at 250 °C based on a solid-liquid-solid reaction. The key to this approach is the catalyst metal, which is not popular for graphene growth by chemical vapor deposition. A catalyst metal film of 500 nm thick was deposited onto an amorphous C (50 nm thick) coated SiO_2_/Si substrate. The sample was then annealed at 250 °C under vacuum condition. Raman spectra measured after the removal of the catalyst by chemical etching showed intense G and 2D peaks together with a small D and intense SiO_2_ related peaks, confirming the transfer free growth of multilayer graphene on SiO_2_/Si. The domain size of the graphene confirmed by optical microscope and atomic force microscope was about 5 μm in an average. Thus, this approach will open up a new route for transfer free graphene growth at low temperatures.

Extraordinary electrical, mechanical and chemical properties of graphene make it suitable for various applications^1^,^2^,^3^,^4^. It has the potential to be used as a future material for post-silicon electronics^5^,^6^. There is also a possibility of molecular scale electronics based on graphene because band gap opening is possible in graphene nano-ribbons^7^,^8^,^9^. There are various techniques for the synthesis of graphene among which chemical vapour deposition (CVD) is popular^10^,^11^,^12^,^13^,^14^. Generally, with CVD high quality, large area and controllable layer numbers of graphene grow on catalytic substrates, such as Cu, at elevated temperatures^15^. However, for a wider range of practical applications, low-temperature growth should be achieved. Thus, much effort has been devoted to the low-temperature graphene growth. In some reports, for example, Marchena *et al*. have achieved direct graphene on flexible glass at 700 °C, Muñoz *et al*. have grown monolayer graphene directly on insulating substrate at 650 °C using plasma assisted CVD, Sulaiman *et al*. demonstrated the CVD graphene growth at 450 °C on Cu by using chlorobenzene as a carbon source. Rummeli *et al*. demonstrated the CVD graphene growth at 325 °C on dielectric insulators using acetylene as a carbon source, although the grown graphene was of sub-micrometer dimension. Jang *et al*. reported graphene formation at 300 °C using benzene using a CVD process^16^,^17^,^18^,^19^,^20^,^21^,^22^. Kwak *et al*. demonstrated the low-temperature growth of graphene film using Ni catalyst^23^, and to attain the continuous graphene showing 2D peak in Raman spectrum, pre-annealing at 1000 °C in H_2_ ambient to yield a strong 111-texture of Ni is necessary. If the low-temperature graphene growth without any high-temperature heat treatment is achieved, it will be very fascinating.

Very recently, we noticed that a variety of metals can be used for graphene growth based on the solid-liquid-solid reaction. In the solid-liquid-solid reaction method, although it is a high-temperature process at the moment, graphene can be grown directly on insulating substrates^24^. So, the transfer process which is usually indispensable for CVD-grown graphene and sometimes hinders the performance of graphene is not needed^25^. If the direct graphene growth on insulating substrates at low temperatures is achievable, it will be fascinating^26^. Here we addressed this subject using tin (Sn), which is not popular in CVD, as a catalyst and amorphous carbon (a-C) as a carbon source in solid-liquid-solid reaction.

## Methods

In the present work, we employed a simple solid-liquid-solid phase reaction method by which graphene grows just by heating of layered films of catalyst metal and carbon on a substrate in a vacuum condition. In this method, the amorphous carbon film can be available as a carbon source, which is advantageous to skip the process of decomposition of carbon-containing molecules by a catalyst, unlike CVD. Pulsed laser deposition (PLD) technique was used to carry out the controlled deposition of a thin C and a catalytic Sn layers. Deposition of 50-nm thickness C layer and 500-nm Sn layer was carried out with a laser pulse energy and frequency of 30 mJ and 10 Hz, respectively, at room temperature. A Si wafer coated with a 100 nm thick SiO_2_ layer (SiO_2_/Si) was used as a substrate for the experiments. Annealing of the Sn/C stack layer on SiO_2_/Si substrate was performed at 250 °C in a PLD system at a pressure of 5 × 10^−5^ Pa. After the annealing, the Sn layer was removed by a chemical etching process using diluted nitric acid (10%) solution. The characterization of graphene sample thus prepared involved optical microscopy (VHX-500 digital microscope), Raman spectroscopy (NRS 3300 laser Raman spectrometer with a laser excitation energy of 532.08 nm), atomic force microscopy (AFM) (JSPM-5200) and Auger electron spectroscopy (AES) (JAMP-7800).

## Results and Discussion

**Figure 1:**
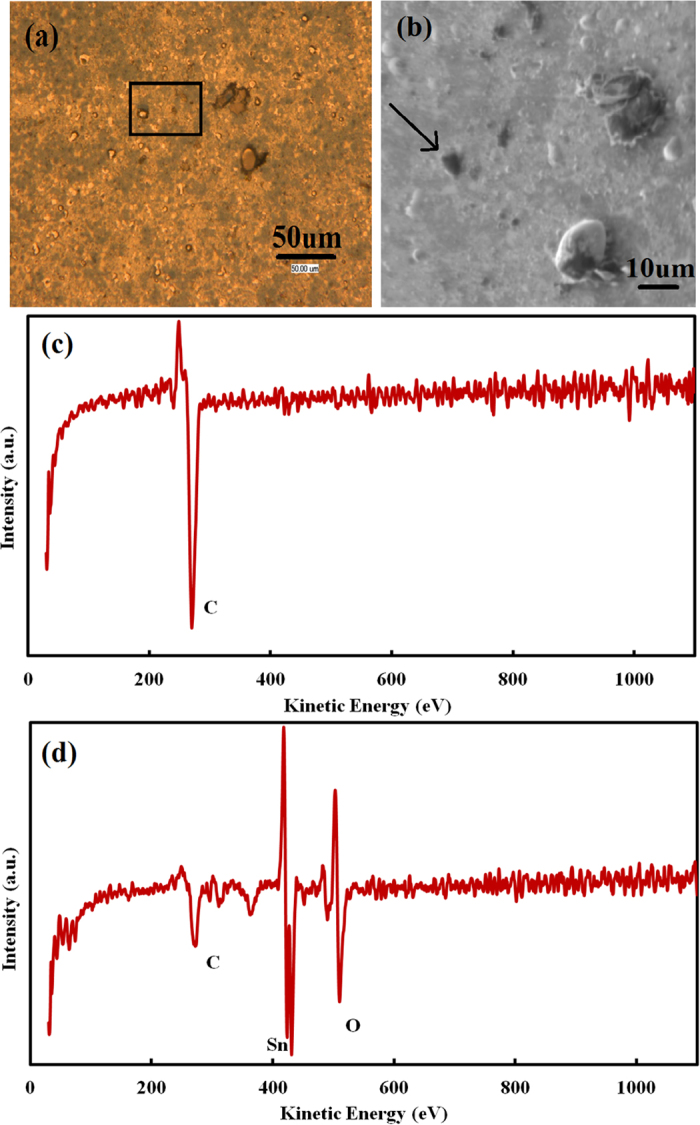
(**a**) Optical image (for a rectangular area in (**a**), see also Fig. 2(a)), (**b**) SEM image of a sample surface after annealing process, (**c**) AES spectrum measured at a dark gray platelet indicated with a black arrow in Fig. (**b**), and (**d**) typical AES spectrum on other places on the sample surface.

Figure 1a shows a typical optical image of a sample surface after an annealing process without chemical etching and Fig. 1b shows a scanning electron microscopy (SEM) image of the same sample surface. On the surface, round-shaped and platelet-like particles which show bright and dark gray contrasts in Fig. 1b, respectively, are dispersed. Elemental composition of the sample surface was investigated using AES. Figure 1c shows an AES spectrum measured at a dark gray platelet indicated with a black arrow in Fig. 1b while Fig. 1d shows a typical AES spectrum of other places on the sample surface. AES investigation indicates that the sample surface, not only round-shape particles but also the ground, consists of C, Sn and O everywhere except dark gray platelets which show only a C peak.

**Figure 2:**
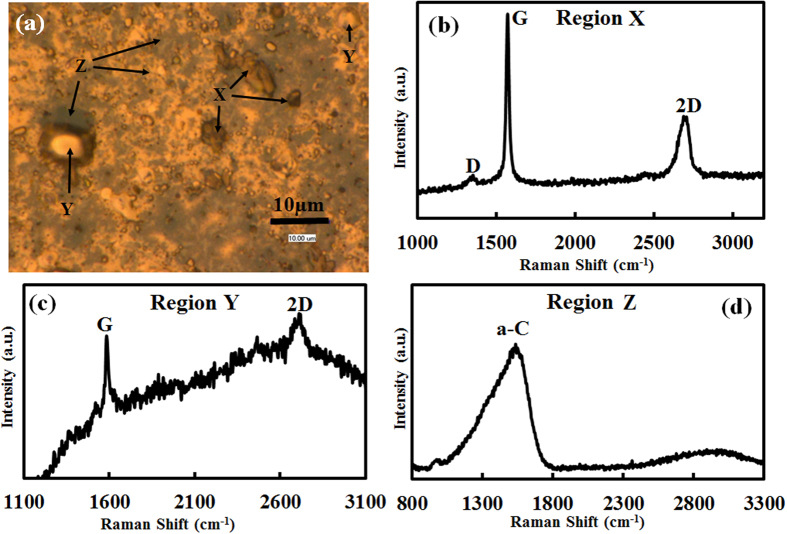
(**a**) Magnified optical image of the rectangular area in Fig. 1(a), (**b–d**) Raman spectra measured at region X, Y and Z in (**a**).

In order to study the carbon state on the sample surface shown in Fig. 1a, Raman analysis was performed. Figure 2a shows a higher magnification image of a rectangular area in Fig. 1a. Figure 2a is labeled with regions X, Y and Z corresponding to dark gray platelet, round-shape particle, and other ground parts in Fig. 2a, respectively. Figure 2b–d displays Raman spectrum at regions X, Y and Z, respectively. Note that the region X and Y show graphene peaks with G and 2D peaks centered at 1583 cm^−1^ and 2711 cm^−1^, respectively, with a less intense D peak at 1354 cm^−1^, while region Z shows an amorphous carbon (a-C) peak. Raman analysis confirms the formation of graphene patches only at regions X and Y surrounded by a-C (Region Z). Thus, it is concluded that graphene grows on Sn even at a temperature as low as 250 °C.

**Figure 3:**
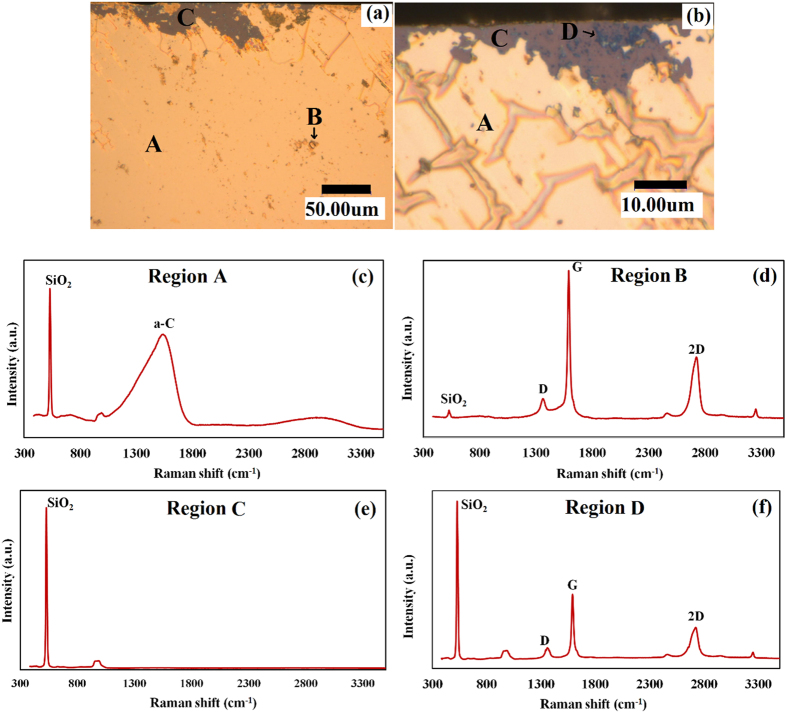
Typical (**a**) low- and (**b**) high-magnification optical images of a sample surface after short time removal of the metal; (**c–f**) Typical Raman spectra obtained at the respective regions A–D in (**a**,**b**).

To confirm whether the formation of graphene occurred directly also on SiO_2_ surface or only on the catalyst metal surface, the sample was kept in 10% nitric acid (HNO_3_) solution for 2 hours (short time) to remove metal from the sample surface. Figure 3a presents a typical low magnified optical microscope image after the short time (2 hours) removal of the metal, on which three regions are clearly visible; in the yellowish ground region (referred as region A hereafter) brown regions (referred as region B hereafter) are dispersed, and gray regions (referred as region C hereafter) are also recognizable at a glance. Figure 3b shows a close-up image of one of the regions A and C near the edge of the sample. In Fig. 3b, contrast is relatively brighter than Fig. 3a. A careful inspection of Fig. 3b reveals that dark blue patches (referred as regions D hereafter) of maximum 5 μm are dispersed in region C.

**Figure 4:**
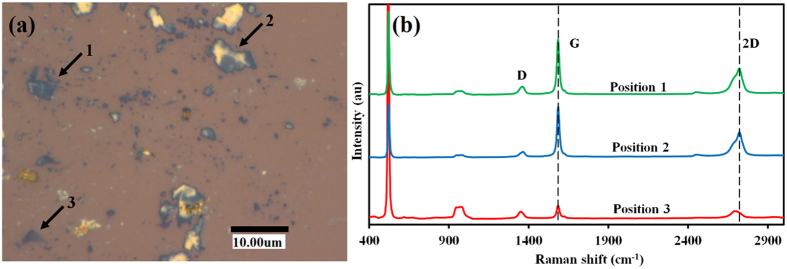
(**a**) Optical image of 5 hours etched sample surface (**b**) Raman spectrum at partially etched positions 1, 2 and completely etched position 3.

In order to identify the carbon state more microscopically, a detailed Raman analysis was performed for the respective regions A–D in Fig. 3a,b. Figure 3c,d show typical Raman spectra for regions A and B, respectively. The Raman spectrum attained at region B revealed intense G and 2D peaks. It should be also noted that multilayer graphene was also formed at region D, while the surrounding region C showed the only SiO_2_ related Raman peak. This result must suggest the evidence of graphene growth directly on the SiO_2_ substrate. Further chemical etching was performed to establish a firm conclusion. Figure 4a shows an optical microscope image taken after chemical etching for 5 hours, between short (2 hours) and long time (24 hours) etching. The yellowish parts are residual Sn and bluish flakes are graphene, and most of another part is the SiO_2_ substrate. As indicated by the arrow labeled with number 2 in the Fig. 4a, some of the residual Sn are on the graphene flakes, which is the evidence of the graphene formation at the Sn/SiO_2_ interface. Raman spectrum measured on position 1, 2 and 3 labeled in Fig. 4a shows that 2D peak shifts towards lower wavenumbers because of the etching process, indicating that graphene layers formed on Sn are being removed along with Sn during etching. Figure 4b shows that the 2D peak for positions 1 and 2 are at 2720 cm^−1^ while that for position 3 is at 2692 cm^−1^. This shift in 2D peak confirms that some layers are removed along with Sn.

**Figure 5:**
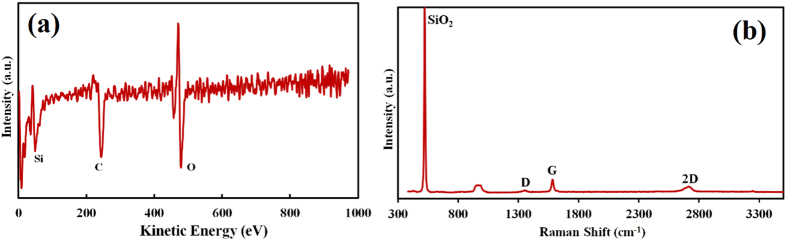
(**a**) AES and (**b**) Raman spectra taken at the dark blue patch corresponding to region D in Fig. 3(b) after a long-time chemical etching.

After the long-time removal of the metal, SiO_2_ substrate was recognizable all over the sample surface and dark blue patches corresponding to region D in Fig. 4b were dispersed thereon. Figure 5a,b show typical AES and Raman spectra taken at the dark blue patch. As seen in the AES spectrum, C and peaks related to substrate SiO_2_ were detected and no metal was observable. As confirmed by the Raman spectrum shown in Fig. 5b, the structure of the carbon was multilayer graphene.

**Figure 6:**
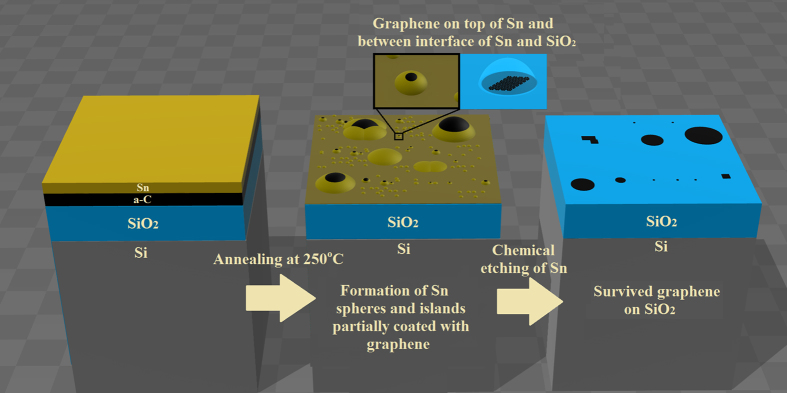
Schematic diagram of annealing and chemical etching process.

From the above-described experimental data, the annealing and etching processes may be summarized as follows together with the schematic representation in Fig. 6: During annealing, sub-micron to about 10 μm Sn particles or flakes in base diameter are formed on the Sn film surface as seen in SEM images shown in Fig. 1a. Sn possesses a catalytic potential to make carbon atoms form the sp^2^ carbon bonding on its surface^27^,^28^,^29^, and the mobility of carbon atoms would increase on the molten Sn surface. Thus, the sp^2^ carbon bonding formation, namely graphene formation, would be enhanced on the molten Sn surface. This graphitization would occur not only at the top surface but also at the interface between Sn and SiO_2_ substrate. After the chemical removal of Sn, the only graphene formed at the interface between Sn and SiO_2_ would have survived on the SiO_2_ surface and the graphene formed on the top of Sn surface would have dispersed in the chemical solution.

**Figure 7:**
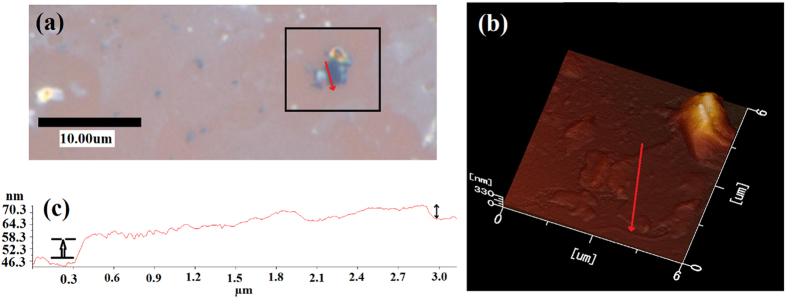
(**a**) Optical image of a sample surface after long time etching showing blue patch. (**b**) AFM image of the rectangle in (**a**,**c**) line profile at the lines indicated by red arrows in (**a**,**b**) for the blue patch.

In order to probe the thickness of the formed graphene, AFM was carried out at around a typical dark blue patch after long-time (24 hours) removal of the Sn metal. Figure 7a,b show an optical micrograph image of the dark blue patch and corresponding AFM image of the enclosed in the rectangular area in Fig. 7a, respectively. Figure 7c shows a line profile taken across the dark blue patch. (Lines in Fig. 7a,b indicate the profiled position.) As seen in Fig. 7c, graphene was of about 5–7 nm in thickness, agreeing with the result of the multilayer graphene concluded earlier by Raman spectra (Figs 3f and 5b).

**Figure 8:**
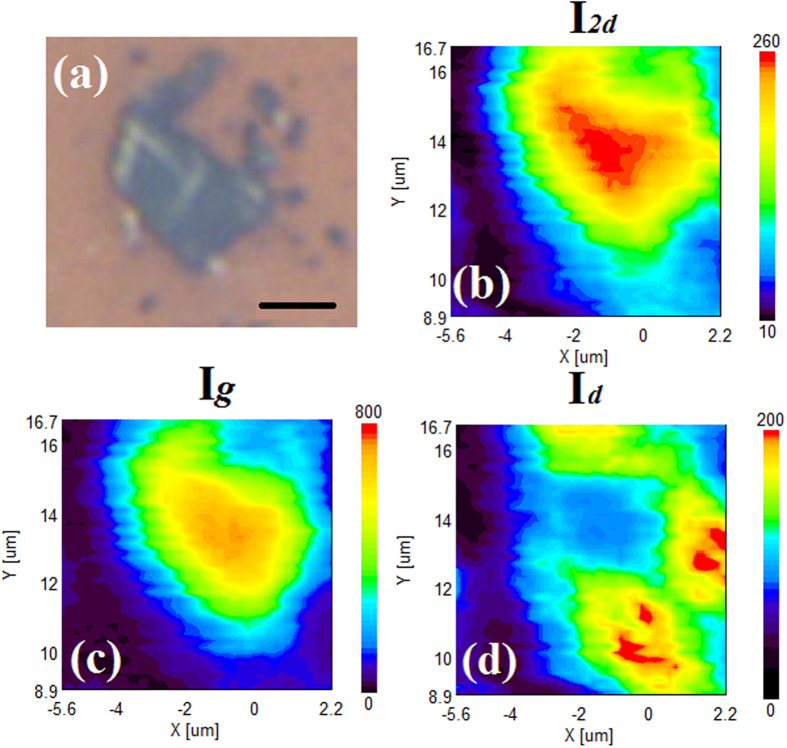
(**a**) Optical image (scale: 2 μm) of graphene flake where Raman mapping is performed. (**b**–**d**) Raman mapping of 2D, G and D peaks, respectively.

In order to confirm the size of the graphene flakes, surface Raman mapping was also performed. Figure 8a shows an Optical image of graphene flake where Raman mapping is performed. Figure 8b–d show Raman mapping of 2D, G and D peaks, respectively. Raman mapping clearly indicates that the graphene layer is spread over a distance of 5 μm. From the mapping it is clearly noticeable that G peak intensity is more than three times the 2D peak intensity, indicating the presence of multilayered graphene layer, complimentary evidence of the above-described AFM data (Fig. 7). Raman mapping of d peak shows that defects are higher near the edges of the graphene layer.

Usually, it is easier to graphitize a-C at higher temperature^30^. To reduce the graphitization temperature, an external stimulating energy aid is needed, which can be provided by adding catalyst^31^,^32^. In most cases a-C dissolves in such catalysts and graphitizes during a cooling process^33^. Catalysts are also considered to move through a-C to form graphitization along their pathway^34^. During this graphitization process, wherever a catalyst moves, it would carry C atoms along with it and thus C atoms would be added or subtracted to form a *sp*^*2*^ hybridized honeycomb arrangement. The reason that *sp*^*2*^ hybridized honeycomb arrangement is preferred compared to other bonding states during catalyst motion through a-C might be due to the fact that *sp*^*2*^ hybridized honeycomb arrangement does not require any extra change in enthalpy to form while *sp*^*3*^ and *sp*^*1*^ do^35^. In the present case due to the solubility of Sn with carbon, although small^29^, carbon atoms would be diffused into Sn and segregate on the surface during thermal annealing^36^, hence forming graphene. Thus, the low-temperature graphene formation based on the solid-liquid-solid phase reaction using low melting metal, Sn, would be achieved as the catalyst.

As described above, although graphene has been easily formed on top of the Sn surface, it has also been formed between the Sn and SiO_2_/Si interfaces. This graphene becomes visible only after the chemical etching process. In order to check the role of substrate in graphene formation, similar experiments replacing SiO_2_ by glass substrates were also performed. It was also the case for glass substrates, thus indicating that graphene would be growable directly on any insulating substrate using this technique. The next step required for this technique is the optimization of the layer thickness of C and Sn and the results will be dealt with in a forthcoming paper.

## Conclusions

In this work, a step towards low-temperature graphene growth using a new catalyst metal, Sn, has been executed and transfer free graphene growth on SiO_2_ substrate at 250 °C has been attempted. Raman microscopy, AES and AFM measurements confirmed the formation of multilayer graphene on SiO_2_ as well as on Sn. Although the formed graphene is still of micrometer order at the moment, this transfer free low-temperature synthesis approach is believed to explore new dimensions of graphene properties and applications.

## Additional Information

**How to cite this article:** Vishwakarma, R. *et al*. Transfer free graphene growth on SiO_2_ substrate at 250 °C. *Sci. Rep.* **7**, 43756; doi: 10.1038/srep43756 (2017).

**Publisher's note:** Springer Nature remains neutral with regard to jurisdictional claims in published maps and institutional affiliations.
